# Serum LDL Promotes Microglial Activation and Exacerbates Demyelinating Injury in Neuromyelitis Optica Spectrum Disorder

**DOI:** 10.1007/s12264-023-01166-y

**Published:** 2024-01-16

**Authors:** Man Chen, Yun-Hui Chu, Wen-Xiang Yu, Yun-Fan You, Yue Tang, Xiao-Wei Pang, Hang Zhang, Ke Shang, Gang Deng, Luo-Qi Zhou, Sheng Yang, Wei Wang, Jun Xiao, Dai-Shi Tian, Chuan Qin

**Affiliations:** 1 Department of Neurology, Tongji Hospital, Tongji Medical College, Huazhong University of Science and Technology, Wuhan, 430030 China; 2 Hubei Key Laboratory of Neural Injury and Functional Reconstruction, Huazhong University of Science and Technology, Wuhan, 430030 China; 3 Department of Neurology, Hainan General Hospital, Hainan Affiliated Hospital of Hainan Medical University, Haikou, 570311 China

**Keywords:** Neuromyelitis optica spectrum disorder, Low-density lipoprotein, Microglia

## Abstract

Neuromyelitis optica spectrum disorder (NMOSD) is an autoimmune inflammatory demyelinating disease of the central nervous system (CNS) accompanied by blood-brain barrier (BBB) disruption. Dysfunction in microglial lipid metabolism is believed to be closely associated with the neuropathology of NMOSD. However, there is limited evidence on the functional relevance of circulating lipids in CNS demyelination, cellular metabolism, and microglial function. Here, we found that serum low-density lipoprotein (LDL) was positively correlated with markers of neurological damage in NMOSD patients. In addition, we demonstrated in a mouse model of NMOSD that LDL penetrates the CNS through the leaky BBB, directly activating microglia. This activation leads to excessive phagocytosis of myelin debris, inhibition of lipid metabolism, and increased glycolysis, ultimately exacerbating myelin damage. We also found that therapeutic interventions aimed at reducing circulating LDL effectively reversed the lipid metabolic dysfunction in microglia and mitigated the demyelinating injury in NMOSD. These findings shed light on the molecular and cellular mechanisms underlying the positive correlation between serum LDL and neurological damage, highlighting the potential therapeutic target for lowering circulating lipids to alleviate the acute demyelinating injury in NMOSD.

**Supplementary Information:**

The online version contains supplementary material available at 10.1007/s12264-023-01166-y.

## Introduction

Neuromyelitis optica spectrum disorder (NMOSD) is an autoimmune-mediated inflammatory demyelinating disease of the central nervous system (CNS). Recurrent attacks have been reported, with the accumulation of severe sequelae such as blindness and weakness in the extremities [1, 2]. Approximately 80% of patients with NMOSD are seropositive for aquaporin-4 immunoglobulin G (AQP4-IgG) [3, 4]. AQP4-IgG binds to AQP4 in astrocytes, triggering the activation of the classical complement cascade reaction, which in turn leads to microglial activation [5–7] and subsequent demyelination [8, 9]. Disruption of the blood-brain barrier (BBB) allows leakage of AQP4-IgG and cytokines, which is considered a critical step in the development of demyelination in NMOSD [10, 11]. Under normal conditions, brain and peripheral lipid metabolism function independently. However, when the BBB is disrupted, lipid components from the peripheral blood can infiltrate into the CNS [12, 13]. Previous studies have shown a significant elevation of triglyceride (TG) and low-density lipoprotein (LDL) levels in the peripheral blood of patients with NMOSD [14]. However, whether these infiltrating lipid species influence the pathogenesis of NMOSD remains to be elucidated.

Microglia are the innate immune cells of the CNS and play a crucial role in the development of NMOSD lesions, including neuroinflammation, BBB disruption, demyelination, and axonal injury [15–17]. Previous studies have shown that LDL promotes the inflammatory response of BV-2 cells [18]. However, the specific role of LDL in microglial activation and the pathogenesis of NMOSD remains uncertain. In this study, we aimed to investigate the impact of LDL on microglial activation during demyelinating injuries and elucidate the underlying mechanisms. We first identified significantly elevated serum LDL in newly diagnosed, untreated NMOSD patients, and this was positively correlated with disease severity. Subsequently, using a mouse model of NMOSD, we confirmed the existence of BBB disruption. We demonstrated that LDL specifically activated microglia and exacerbated demyelination. In addition, we further investigated the mechanism underlying LDL-induced microglial activation. Our findings revealed that serum LDL targeted microglia in the CNS and modulated their phenotypic and functional changes and serves as a potential therapeutic target in NMOSD.

## Materials and Methods

### Participants and Sample Collection

The study was approved by the Ethics Committee of Tongji Hospital, Huazhong University of Science and Technology (Ethics Approval Number: TJ-IRB20190502). Patients with AQP4-IgG seropositive NMOSD (confirmed by cell-based assay) admitted to Tongji Hospital and who met the diagnostic criteria published in 2015 [19] were prospectively enrolled after signing an informed consent form. Only newly diagnosed and untreated patients were included. Patients who had ever received lipid-lowering therapy were also excluded. Clinical data, including gender, age, the Expanded Disability Status Scale (EDSS) score, and serum lipid levels, were also collected. Serum samples were centrifuged immediately after collection and stored at − 80 °C until further use.

### Serum Neurofilament light (NFL) Measurement

NFL in serum was detected using an electrochemiluminescence immunoassay (Cat#F217X, Meso Scale Discovery).

### Purification of AQP4-IgG

Serum from 4 AQP4-IgG-positive NMOSD patients was collected according to a previous study [20]. IgG was purified with G-agarose glycoprotein, and the concentration of IgG was adjusted to 20 mg/mL, referred to as AQP4-IgG. Clinical information of these NMOSD patients is shown in Additional file 2: Table S1.

### Establishment of the Mouse NMOSD Model

Animal care and experimental procedures were carried out in accordance with the institutional guidelines and approved by the Ethics Committee of Tongji Hospital, Huazhong University of Science and Technology (Ethics Approval Number: TJH-202201008). Wild-type (WT) C57BL/6 mice were used. Female mice were used in the experiments primarily as a quality control strategy to reduce the effect of sex on NMOSD. No animals were excluded. No randomization was performed. Microglial morphology was analyzed using a blind method. No statistical assessment was performed to determine the sample size, but the sample size for each experiment was similar to our previous studies [20]. All mice were placed in a room at an appropriate temperature (24 °C–25 °C) with a 12-h light-dark cycle. Food and water were freely available. Mice were anesthetized with isoflurane (induction: 3% isoflurane in O_2_ at 0.3 L/min; maintenance: 1.5% isoflurane in O_2_ at 0.3 L /min) and placed on a stereotaxic frame as previously described [20, 21]. After the toe irritation response disappeared, the scalp was cut to expose the bregma. A single hole was drilled 2 mm to the right of the bregma with a high-speed drill. A 33 G needle was attached to a 25 μL glass syringe, inserted 3 mm deep, and injected with 6 μL AQP4-IgG and 4 μL human complement (HC) (a total of 10 μL) at a rate of 0.5 μL/min. After the operation, the wound was sutured with 4-0 nylon and sterilized by iodophor. For LDL or Dil-LDL treatment, 100 μg/mL LDL was mixed with AQP4-IgG and HC and injected into the striatum. For evolocumab (a PCSK9 inhibitor) treatment, 10 mg/kg evolocumab was injected subcutaneously after AQP4-IgG and HC injection.

### Modified Neurological Severity Score (mNSS)

Neurological function was assessed using the mNSS test at 1, 3, and 7 days after NMOSD. It consisted of motor (muscle status, abnormal movement), sensory (visual, tactile, and proprioceptive), reflex, and balance tests with scores ranging from 0 to 18 with higher scores indicating more severe neurological deficits [22].

### Balance Beam

As previously described [23], the balance beam apparatus mainly consisted of a 1 m long and 6 mm wide balance beam placed on a 50 cm high platform with a cushioning device underneath. Mice were placed on one side of the beam. They were trained to successfully cross the entire beam. The training lasted for two days. On the day of the test, the time to pass across the beam was recorded. Before placing the next mouse, the apparatus was wiped with a towel soaked in 75% ethanol.

### Preparation of Brain Frozen Sections

After completing behavioral tests, mice were anesthetized with pentobarbital (50 mg/kg) and transcardially perfused with 30 mL of cooled PBS, followed by 30 mL of cooled 4% paraformaldehyde. After perfusion, brains were harvested and post-fixed in 4% PFA overnight at 4 °C, and then completely dehydrated with 30% sucrose. Serial coronal sections were cut at 20 μm on a freezing microtome (CryoStar, NX50, Thermo).

### HE Staining

Brain sections were stained with hematoxylin for 5 min and counterstained with eosin for 3 min. The stained sections were treated sequentially with 95% ethanol, anhydrous ethanol, and xylene. Images were captured under a light microscope (Olympus).

### Oil Red O Staining

Brain sections were rinsed with 60% isopropanol for 30 s, stained with freshly prepared ORO solution at room temperature for 10 min, and then washed with 60% isopropanol to remove excess dye. Images were captured under a light microscope (Olympus).

### Luxol Fast Blue (LFB) Staining

Brain sections were dehydrated in gradient alcohols and soaked in 0.1% LFB at 60 °C for 6 h–8 h. The sections were then rinsed in 0.05% lithium carbonate and 70% alcohol until the myelin was blue. Images were captured under a light microscope (Olympus) and the area of demyelinating lesions was assessed using ImageJ (NIH).

### Western Blot

Briefly, 500 μL of 1% neutral red solution was injected intraperitoneally ~ 2 h–3 h before brain extraction to better differentiate demyelinating lesions from relatively healthy tissue [24, 25]. Isolated tissue was thoroughly homogenized in lysis buffer supplemented with phosphatase inhibitors. Lysates were centrifuged at 12,000 g for 15 min at 4 °C. The supernatant was collected and the protein concentration was determined by the BCA method. Total protein (20 μg–40 μg) was added to 10% sodium dodecyl sulfate-polyacrylamide gels and adsorbed onto 0.45 μmol/L nitrocellulose (NC) filter membranes. Proteins were blocked with 5% skim milk for 1 h at room temperature and then incubated with primary antibody overnight. NC membranes were rinsed three times with tris-buffered saline containing 0.05% Tween-20 and incubated with horseradish peroxidase-labeled secondary antibody for 1 h at room temperature. A complete list of the antibodies used is shown in Additional file 2: Table S2. Exposures were made using chemiluminescent reagents and captured with a CCD camera (BLT, GelView 6000pro).

### Microglia and Oligodendrocyte Progenitor Cell (OPC) Cultures

Primary microglia and OPC cultures were prepared from mice using the differential attachment method [26, 27]. Briefly, initial mixed glial cell cultures were obtained from the brains of WT P1–2 neonates. Isolated cells were cultured in poly-dimethyl lysine (PDL) coated flasks containing 20% fetal bovine serum (FBS) in DMEM/F12. After cells were cultured in a humidified incubator at 37 °C and 5% CO_2_ for 3 days, the medium was changed to DMEM/high glucose containing 20% FBS, and the upper layer of microglia was collected after 9 days–12 days. After removing the microglia, the remaining cells were digested with 0.25% trypsin and OPCs were isolated from the astrocyte layer. The cell suspension was transferred to a dish and incubated for 30 min to allow the astrocytes and microglia to adhere to the surface while the OPCs remained in suspension. The OPCs were then plated on PDL-coated plates and maintained for 3 days–5 days in serum-free basal medium (DMEM/F12, 0.1% bovine serum albumin, 1% N2, 2% B27) containing 10 ng/mL platelet-derived growth factor and 10 ng/mL basic fibroblast growth factor to induce oligodendrocytes. OPCs were stimulated with T3 (50 ng/mL) and ciliary neurotrophic factor (10 ng/mL) to stimulate OPCs or co-cultured with treated microglia for 3 days. Co-cultured microglia were pretreated with or without LDL, and then the medium in the co-culture system was replaced with fresh DMEM before co-culturing with OPCs.

### Myelin Purification

Myelin was purified from adult WT C57BL/6 mice brains as previously described [28, 29]. Brains were placed in cold lysis buffer (pH 7.4, 10 mmol/L HEPES, 5 mmol/L EDTA, 0.3 mol/L sucrose, and the protease inhibitor phenylmethylsulfonyl fluoride) and homogenized. After transferring the homogenate to a polypropylene tube, an equal amount of 0.32 mol/L sucrose was carefully added underneath the homogenate layer, followed by an equal amount of 0.85 mol/L sucrose below the two layers. The samples were centrifuged in an Optima XPN-100 SW41Ti rotor supercentrifuge (Beckman) at 75,000 g for 30 min at 4 °C with low acceleration and deceleration. Myelin was carefully removed from the 0.32/0.85 mol/L sucrose interface, resuspended in 10 mL distilled water, and centrifuged at 75,000 g, at 4 °C for 15 min with maximum acceleration and deceleration. The pellet was resuspended in 10 mL of distilled water and the above centrifugation procedure was repeated twice. Finally, the pellet was resuspended in lysis buffer and the above steps were repeated to obtain pure myelin. After purification, the myelin was resuspended in PBS and adjusted to a concentration of 1 mg/mL using the BCA Protein Assay Kit. Myelin was labeled with carboxyfluorescein succinimidyl ester (HY-D0938; MCE, NJ, USA) according to the manufacturer's instructions.

### Reagent Treatment

Microglia were treated with 200 ng/mL lipopolysaccharide (LPS), 5 μg/mL myelin, and 50 μg/mL LDL to co-stimulate microglia for 8 or 24 h, and LDL was replaced with PBS in the control group.

### Immunofluorescence

The samples were permeabilized with 0.25% Triton-X100 for 10 min. After blocking for 15 min at room temperature, the samples were incubated with primary antibody overnight at 4 °C, then incubated with secondary antibody for 1 h at room temperature in the dark, and finally sealed with an anti-fluorescent quenching agent containing 4′,6′-diaminocyano-2-phenylindole (DAPI). Images were captured with a confocal microscope (FV1200, Olympus) for further analysis. A complete list of antibodies used is shown in Additional file 2: Table S2.

For brain sections, 4–5 high magnification fields or 1–2 low magnification fields were randomly selected in the lesion area and images were analyzed for each sample. The criterion used to determine the lesion area was to identify regions of interest (ROIs) located along the border between the normal and damaged areas. Positive cells were electronically labeled with software to avoid double counting. To quantify microglial morphology *in vivo*, z-stack images of brain sections (800 × 800 pixels) were obtained on a confocal microscope using a 60× oil-based objective. Images were analyzed semi-automatically using Imaris. Iba1^+^ microglial morphology was analyzed according to a previously described method [30]. Images were analyzed using the maximum intensity projection method, and 10–15 microglia per mouse were analyzed. The area and volume of the microglial soma were determined using the surface rendering method of Imaris.

For BODIPY staining, microglia were plated on PDL-coated glass coverslips, then the coverslips were collected, fixed with 4% PFA for 15 min, and washed three times with PBS. After incubation with primary and secondary antibodies, cells were incubated with BODIPY 493/503 (1:1000 in PBS, ThermoFisher) for 15 min at room temperature. The fluorescence was calculated as the intensity of BODIPY per unit area.

### RT-PCR

Total microglial RNA was extracted with TRIzol. cDNA was reverse transcribed from 1 μg RNA using PrimeScript™ RT Master Mix (Takara) according to the manufacturer's instructions. qPCR was applied using Hieff qPCR SYBR Green Master Mix (Yeasen). All reactions were performed using a real-time PCR system (CFX96, BioRad). The specificity of each reaction was determined by melting curve analysis. The expression of target genes was normalized to β-actin and calculated by the 2^−ΔΔCt^ method. A complete list of primers is shown in Additional file 2: Table S3.

### Statistical Analysis

For clinical data, the Pearson χ^2^ test was used to compare categorical variables between the two groups. Continuous variables were analyzed using the Mann-Whitney U test. The correlation between serum LDL and NFL or the EDSS was calculated using Spearman correlation analysis. For animal data, unpaired Student's t-tests with two-tailed distributions and one-way ANOVA with Bonferroni multiple comparisons were applied. Generalized linear models were used to analyze the mNSS. *P*-values < 0.05 were considered significant. SPSS Statistics 21 and GraphPad Prism 8 were used for statistical analysis.

## Results

### Serum LDL is Correlated with Neurological Damage in NMOSD

**Fig. 1:**
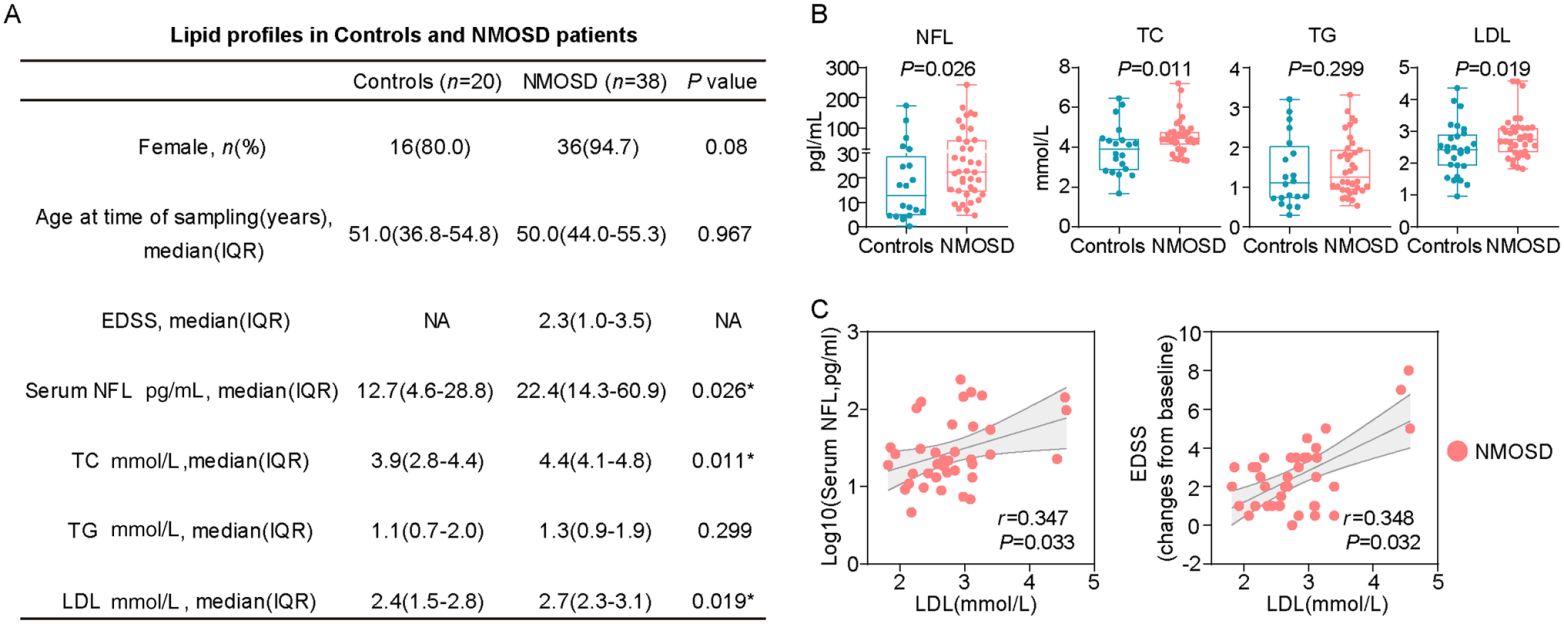
Serum LDL is Correlated with the Neurological Damage in NMOSD. **A** Clinical characteristics and biomarkers in the cohort of AQP4-IgG seropositive NMOSD patients and controls. Pearson’s χ^2^ test was applied to compare the categorical variables between groups. The Mann-Whitney test was applied to compare the continuous variables. *P* values <0.05 are considered significant. **B** Boxplots showing serum NFL, TC, TG, and LDL in AQP4-IgG seropositive NMOSD patients and controls. *P* values are assessed by the Mann-Whitney test. **C** Scatter plots representing the association between serum LDL and NFL or ΔEDSS among AQP4-IgG seropositive NMOSD patients. Spearman analysis was used for statistical correlation. Correlation coefficients (*r*) and *P* values are shown.

We first analyzed the serum lipid levels in a cohort of NMOSD patients. Considering the possible effects of immunotherapy on glycolipid metabolism, only those patients with new-onset, AQP4-IgG seropositive NMOSD during acute attacks, without administration of steroids, immunomodulatory therapy, and lipid-lowering medication were enrolled. The baseline characteristics of the patients are shown in Fig. 1A. There were no statistically significant differences in age and sex between the NMOSD patients and controls. Compared to controls, NMOSD patients had significantly higher total cholesterol (TC) and LDL levels. Notably, the serum NFL (a marker of neurological damage) was also significantly higher in NMOSD patients (Fig. 1B). As LDL is the primary carrier of cholesterol in the blood, we further examined the correlation between serum LDL and NFL or the EDSS change from baseline (ΔEDSS, a marker of neurological deterioration). As expected, serum LDL was positively correlated with serum NFL (*r* = 0.347, *P* = 0.033) and ΔEDSS (*r* = 0.348, *P* = 0.032) (Fig. 1C). These results collectively support the association between serum LDL levels and disease progression in NMOSD.

### Blood-Brain Barrier is Disrupted in the Mouse NMOSD Model

**Fig. 2:**
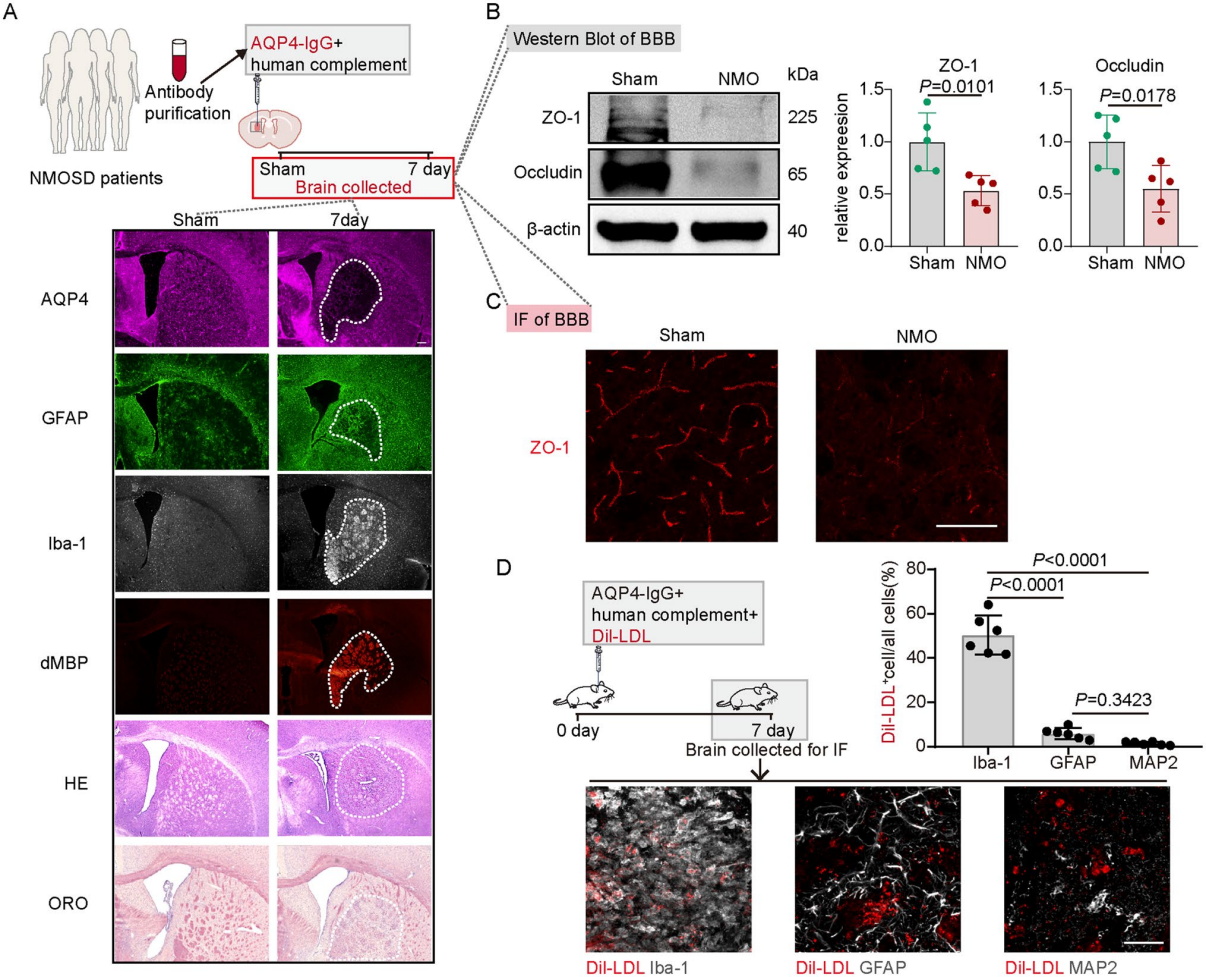
Blood-Brain Barrier is Disrupted in the Mouse NMOSD Model. **A** Schematic of the establishment of the mouse NMOSD model. Representative images of HE staining, ORO staining, AQP4, GFAP, Iba-1, and dMBP immunostaining at 7 days after AQP4-IgG and HC injection. Scale bar, 200 μm. **B** Representative western blots and quantification of the relative expression of ZO-1 and Occludin in sham and NMO groups. *n* = 5, mean ± SD, one-way ANOVA followed by Bonferroni’s *post hoc* test. The full membrane of WB is included in Fig. S1. **C** Representative images of ZO-1 immunostaining in sham and NMO groups. Scale bar, 100 μm. **D** Representative images and quantification of Dil-LDL co-immunostaining with Iba1, GFAP, and MAP2. *n* = 6, mean ± SD, one-way ANOVA followed by Bonferroni’s *post hoc* test. Scale bar, 50 μm.

To further investigate our hypothesis that elevated serum LDL exacerbates NMOSD-related damage, we established a mouse NMOSD model in which AQP4-IgG containing HC was injected into the striatum of WT mice. By immunofluorescence staining, we found a significant loss of AQP4 within the lesion areas at 7 days post injection (dpi), accompanied by microglial activation (a marker of microglia, ionized Ca^2+^-binding adapter molecule 1, Iba-1^+^), astrocytosis surrounding the lesion (GFAP, a marker of astrocytes), and demyelination (a marker of degraded myelin basic protein, dMBP^+^), consistent with our previous study [31]. HE staining and Oil Red O staining also revealed inflammatory cell infiltration and substantial lipid deposition in the demyelinating areas (Fig. 2A).

We then investigated the integrity of the BBB in the mouse NMOSD model. Western blots showed that ZO-1 and Occludin protein (markers of BBB integrity) were significantly decreased after AQP4-IgG and HC injection (Fig. 2B). Consistent with the western blot results, immunofluorescence staining also showed a remarkable decrease of ZO-1 protein in the lesion areas (Fig. 2C). Then, we mixed Dil-labeled LDL with AQP4-IgG, and directly injected them into the striatum. Subsequent results revealed that Dil-LDL was specifically elevated in microglia in the NMOSD model compared to astrocytes and neurons (MAP2 as a marker of neurons) (Fig. 2D). These data suggest that the BBB was disrupted in the mouse NMOSD model, which allowed lipid in peripheral blood to infiltrate into the demyelinating lesions and eventually activate microglia.

### LDL Injection Aggravates Demyelination and Promotes Microglial Activation in the Mouse NMOSD Model

**Fig. 3:**
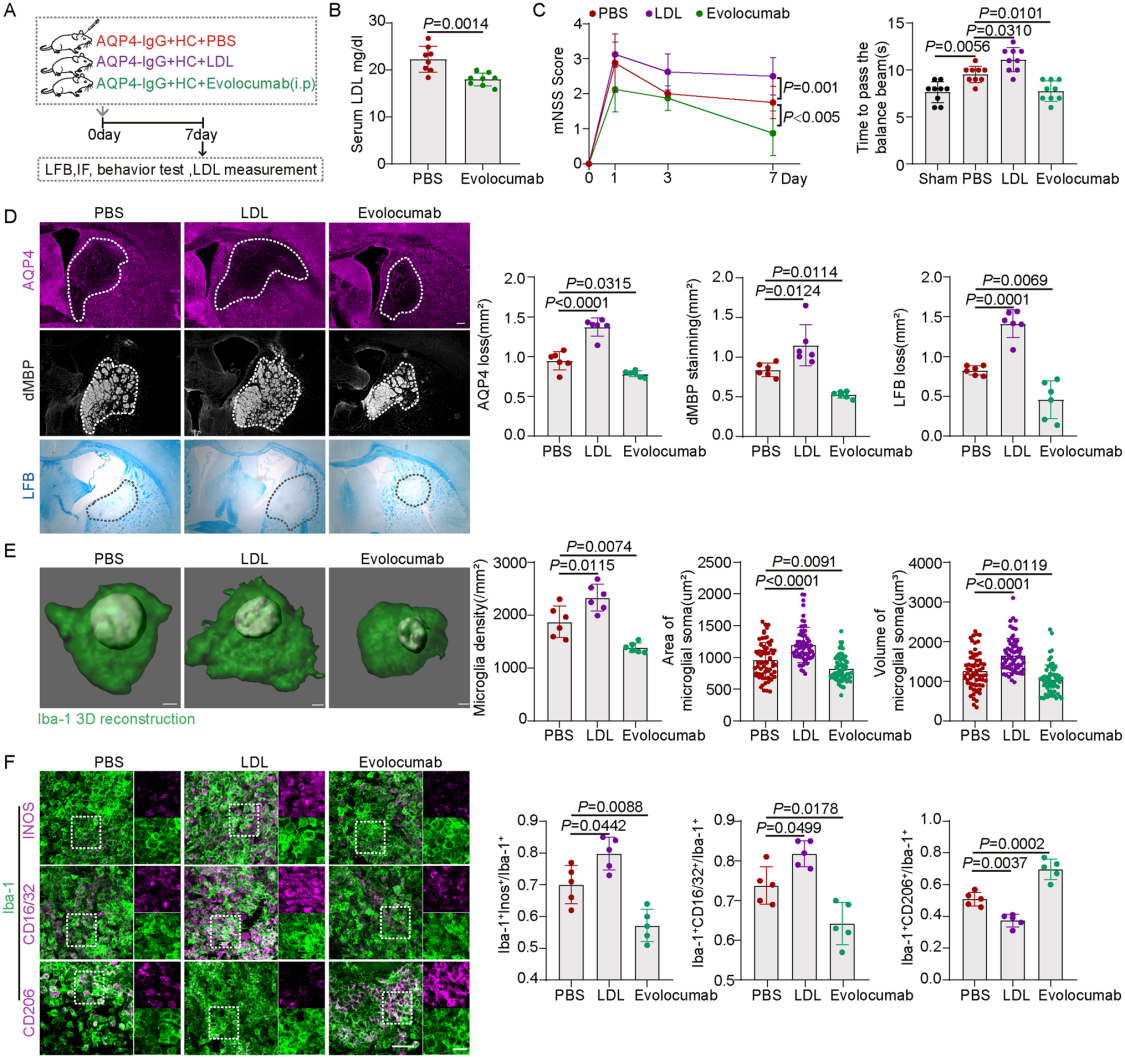
LDL Aggravates Motor Function, Demyelination, and Microglial Activation in Mice. **A** Schematic of PBS, LDL, and evolocumab treatment in the mouse model of NMOSD. **B** Quantification of serum LDL after evolocumab treatment in mice. *n* = 8, mean ± SD, unpaired *t-*test. **C** mNSS scores in PBS, LDL, and evolocumab groups at 1, 3, and 7 days after AQP4-IgG and HC injection. *n* = 8, mean ± SD, generalized linear models. Time to cross the balance beam in Sham, PBS, LDL, and evolocumab groups at 7 days after AQP4-IgG and HC injection. *n* = 9, mean ± SD, one-way ANOVA followed by Bonferroni’s post hoc test. **D** Representative images and quantification of LFB staining, AQP4, and dMBP immunostaining. *n* = 4–6, mean ± SD, one-way ANOVA followed by Bonferroni’s post hoc test. Scale bar, 200 μm. **E** Representative images of Iba-1 immunostaining after 3D reconstruction. Quantification of Iba-1^+^ density, Iba-1^+^ cell soma area, and volume. *n* = 6, 14 microglia per mouse, mean ± SD, one-way ANOVA followed by Bonferroni’s post hoc test. Scale bar, 2 μm. **F** Representative images and quantification of INOS, CD16/32, CD206 and Iba-1 co-immunostaining. *n* = 5, mean ± SD, one-way ANOVA followed by Bonferroni’s post hoc test. Scale bar, 50 μm, and 20 μm (enlarged images).

To understand the effect of LDL on demyelination, we next directly injected LDL into the striatum of WT mice along with AQP4-IgG and HC (Fig. 3A). Compared with the PBS group, mice after LDL injection had higher mNSS scores and took longer to cross the balance beam (Fig. 3C). Also, the lesion areas became larger after LDL injection compared to the PBS group by immunofluorescence staining of AQP4 and dMBP, as well as LFB staining (Fig. 3D).

We next determined whether LDL affected the microglial activation in NMOSD by investigating the density and morphological changes of microglia. Staining for Iba-1 showed that microglia were significantly aggregated in the lesion areas after LDL injection and the soma of microglia was larger (Fig. 3E). In addition, we explored the effect of LDL on the phenotypic transformation of microglia. Fc RII/III receptors (CD16/32) and inducible nitric oxide synthase (iNOS) are markers of classically-activated microglia, while CD206 is a marker of alternatively-activated microglia. The percentage of CD16/32 or iNOS and Iba-1 co-immunostaining was significantly increased and the percentage of CD206 and Iba-1 co-immunostaining was decreased after LDL injection compared to the PBS group (Fig. 3F). These results suggested that LDL exacerbated demyelinating damage and promoted classic microglial activation.

### Evolocumab Rescues Demyelination and Inhibits Microglial Activation in the Mouse NMOSD Model

Evolocumab, a proprotein convertase subtilisin/kexin type 9 (PCSK9) inhibitor, is a strong LDL-lowering drug in clinical use. PCSK9 is a secreted protein that binds and transports the low-density lipoprotein receptor (LDLR) into the lysosome for degradation. Evolocumab works by blocking the binding of PCSK9 to LDLR and leads to LDL reduction [32–34]. Our study showed a decrease in serum LDL in mice at 7 days after administration of evolocumab (Fig. 3A, B). Mice treated with evolocumab had lower mNSS scores and took less time to cross the balance beam than the PBS group (Fig. 3C). The results also showed that evolocumab intervention significantly reduced the lesion areas (Fig. 3D). Moreover, in the evolocumab group compared with the PBS group, fewer microglia were aggregated and the soma was smaller (Fig. 3E). The proportion of CD16/32 or iNOS and Iba-1 co-immunostaining was significantly decreased after evolocumab treatment, while the proportion of CD206 and Iba-1 co-immunostaining was increased (Fig. 3F). In short, we revealed that evolocumab alleviated demyelination and inhibited microglial activation by lowering serum LDL.

### LDL Exacerbates Demyelination by Regulating the Activation and Glycolipid Metabolism of Microglia

**Fig. 4:**
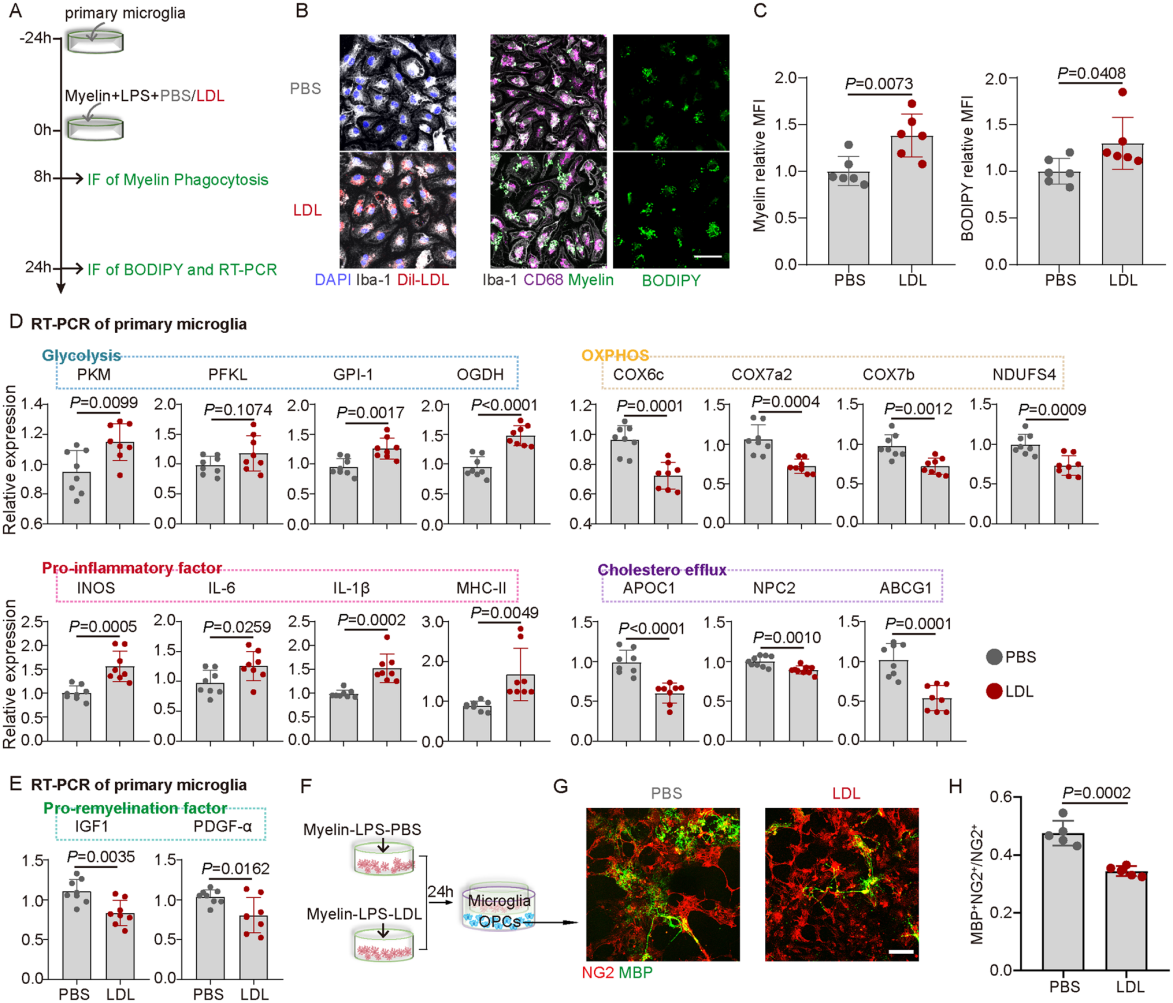
LDL Exacerbates Demyelination by Regulating the Activation and Glycolipid Metabolism of Microglia. **A** Schematic of primary microglia treatment with LDL. **B** Representative images of LDL and Iba-1 co-immunostaining, myelin, CD68, and Iba-1 co-immunostaining, and BODIPY staining with or without LDL treatment. Scale bar, 50 μm. **C** Relative mean fluorescence intensity (MFI) of myelin and BODIPY with or without LDL treatment. *n* = 6, mean ± SD, unpaired *t-*test. **D** RT-PCR of glycolysis, OXPHOS, pro-inflammation, and cholesterol efflux-related gene expression with or without LDL treatment. *n* = 8, mean ± SD, unpaired *t-*test. **E** RT-PCR of pro-remyelination related gene expression with or without LDL treatment. *n* = 8, mean ± SD, unpaired *t-*test. **F** Schematic of co-culture of microglia and OPCs. **G** Representative images of NG2 and MBP co-immunostaining with PBS-treated or LDL-treated microglia. **H** Quantification of NG2^+^MBP^+^/NG2^+^ with PBS-treated or LDL-treated microglia. *n* = 5, mean ± SD, Unpaired *t*-test. Scale bar, 50 μm.

To further explore the potential mechanisms underlying how LDL exacerbates demyelination and microglial activation, we treated primary microglia with LPS and myelin debris *in vitro* to simulate microglia in the inflammatory and demyelinating microenvironment of NMOSD (Fig. 4A). Under this premise, LDL treatment was administered and a higher accumulation of LDL was then found in microglia. Compared to the PBS group, LDL-treated microglia phagocytosed more myelin debris (8 h after treatment) but were unable to degrade it sufficiently (24 h after treatment) (Fig. 4B, C). At 24 h after LDL treatment, microglia showed increased expression of the "glycolysis" genes and decreased expression of the "OXPHOS" genes. These results suggested a shift in energy requirement from OXPHOS to glycolysis. Meanwhile, LDL treatment increased the expression of microglial inflammatory genes and decreased cholesterol efflux as well as the expression of pro-regeneration genes (Fig. 4D, E). Furthermore, it has been found that the process of remyelination plays a fundamental role in the treatment of demyelinating diseases [35]. We then tested the effects of LDL-treated microglia on oligodendrocyte differentiation using a co-culture system. The results demonstrated that microglia treated with LDL inhibited the differentiation of NG2^+^ oligodendrocytes into MBP^+^ mature oligodendrocytes, as compared to microglia treated with PBS (Fig. 4F–H). Our results suggest that LDL exacerbates demyelination by modulating microglial immunometabolic homeostasis.

## Discussion

Although several studies have previously established the role of microglia in NMOSD [36, 37], studies on the modulation of microglia by circulating lipids are still lacking. NMOSD is an inflammatory demyelinating disease, with myelin comprising a diverse range of lipids. Therefore, the equilibrium of lipid metabolism may present a novel target for NMOSD treatment. In this study, we present a comprehensive analysis of clinical patient data and a mouse NMOSD model to elucidate the effect of serum LDL on the exacerbation of demyelination. Moreover, we further showed that serum LDL promotes demyelination through microglial activation and the disruption of glucolipid metabolism.

Indicators for evaluating the activity and progression of NMOSD include clinical markers like EDSS and neural injury biomarkers such as NFL. Following axonal injury, NFL proteins are released into the cerebrospinal fluid, traverse the leaky BBB, and can be detected in the peripheral blood [38, 39]. Elevated serum NFL levels generally indicate CNS axonal injury. Similar to previous studies [14], we found that serum TG, LDL, and NFL were higher in NMOSD patients than in controls. Interestingly, we identified a robust positive correlation between serum LDL and changes in both EDSS and NFL, strongly suggesting that serum LDL is associated with NMOSD disease activity and progression.

Moreover, in line with the pathology of NMOSD, we established a mouse NMOSD model. The results showed that LDL in the brain parenchyma activated microglia, and further exacerbated demyelination. Remarkably, the activation of microglia was effectively inhibited by lowering serum LDL using the PCSK9 inhibitor evolocumab. These results provide further evidence that serum LDL has an impact on the phenotypic transformation of microglia in the CNS, which is in accord with a study in cardiac ischemia/reperfusion injury [33].

*In vitro*, excessive myelin debris and lipid droplets accumulated in microglia after LDL treatment. Remarkably, the process of myelin phagocytosis by microglia is a double-edged sword. On the one hand, the removal of myelin debris aids in facilitating myelin regeneration. However, on the other hand, the excessive accumulation of myelin debris leads to microglial dysfunction and ultimately hinders the degradation of debris [40, 41]. PCR analysis further revealed that LDL promoted microglial activation and glycolysis while inhibiting pro-remyelinating factors and cholesterol efflux. Co-culture experiments with OPCs showed that LDL-treated microglia inhibited the maturation of OPCs. These findings align with previous studies on aging and white matter damage, where lipid-rich myelin debris induces microglia to produce large amounts of pro-inflammatory cytokines [42]. It is known that metabolic reprogramming underlies the phenotypic transformation of microglia [43, 44]. Considering that LDL is a lipoprotein rich in cholesterol, therapies targeting LDL may prove to be an effective strategy for inflammatory demyelinating diseases of the CNS. Despite these findings, additional cellular experiments are still required to explore the specific mechanisms involved. Longer times should be used to investigate the amount of myelin phagocytosis and degradation by microglia after LDL treatment. Previous studies have demonstrated that continuous AQP4-IgG injection into the spinal subarachnoid space induces demyelination, and this may offer potential strategies for clinical treatment and warrant further investigation.

**Fig. 5:**
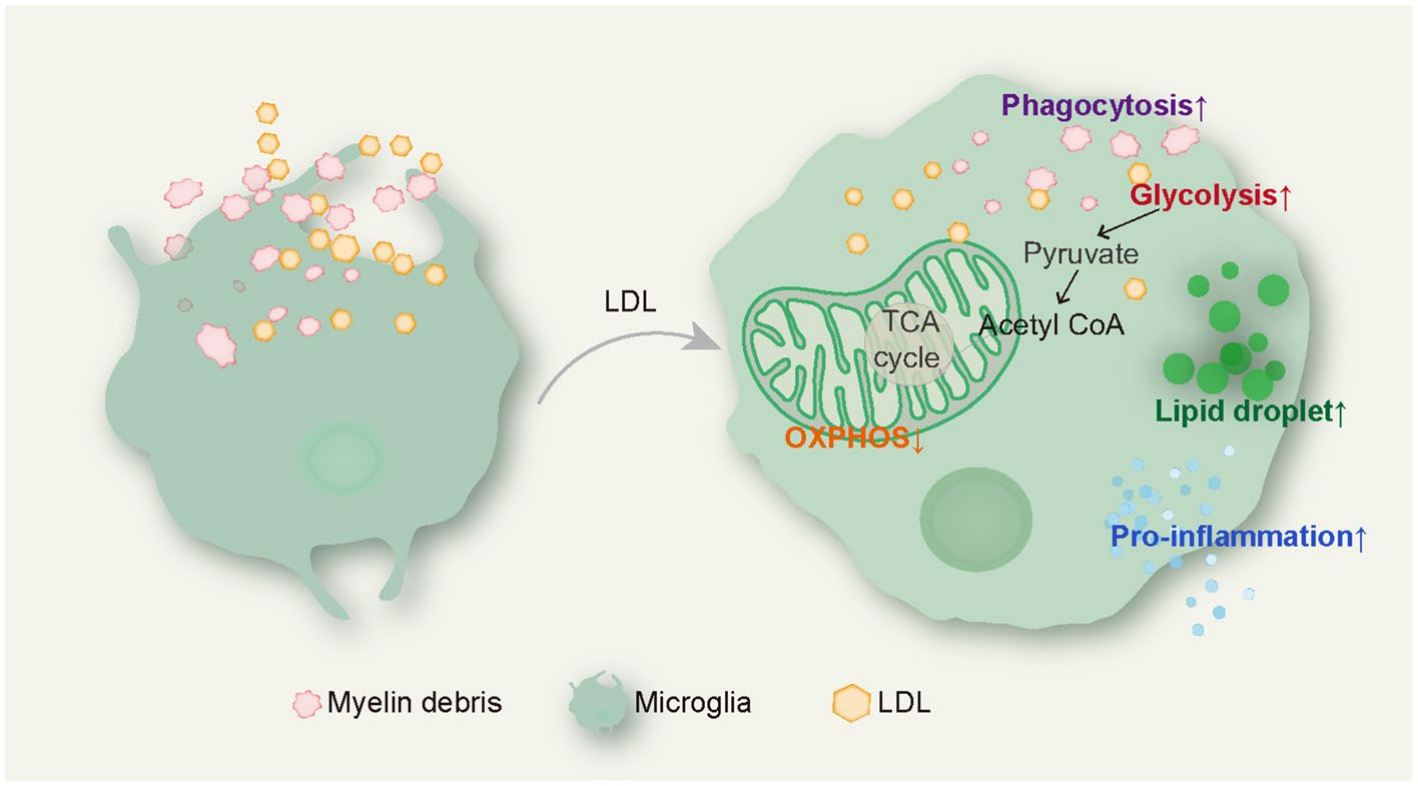
Schematic Representation of the Mechanism of Microglia Activation by LDL. Microglia are over-activated to secrete pro-inflammatory cytokines in response to LDL stimulation; they contain large amounts of myelin debris and lipid droplets. Moreover, dysfunctional microglia exhibit an enhancement of glycolysis and inhibition of oxidative phosphorylation.

In conclusion, our study revealed a correlation between serum LDL and disease severity in NMOSD patients based on a clinical cohort. The mouse NMOSD model provided further evidence of LDL leakage into demyelinating lesions *via* a leaky BBB. Once inside the CNS, LDL was phagocytosed by microglia, leading to an exacerbation of demyelination by promoting microglial activation and dysregulating glycolipid metabolism (Fig. 5). Collectively, our data suggest that therapies aimed at lowering circulating LDL may serve as an effective intervention for the acute demyelination in NMOSD.

### Supplementary Information

Below is the link to the electronic supplementary material.

Supplementary file1 (PDF 248 KB)

## Data Availability

The datasets generated during and/or analyzed during the current study are available from the corresponding author upon reasonable request.
