# Supplementary material for: Serum LDL Promotes Microglial Activation and Exacerbates Demyelinating Injury in Neuromyelitis Optica Spectrum Disorder
Source: Neurosci Bull. 2024 Jan 16;40(8):1104–14. doi: 10.1007/s12264-023-01166-y (PMC11306683; doi:10.1007/s12264-023-01166-y)
Supplement: Supplementary file 1 — Supplementary file1 (PDF 248 KB) [file 12264_2023_1166_MOESM1_ESM.pdf]

Supplementary Materials

Fig. S1 Full membrane of WB for Fig. 2

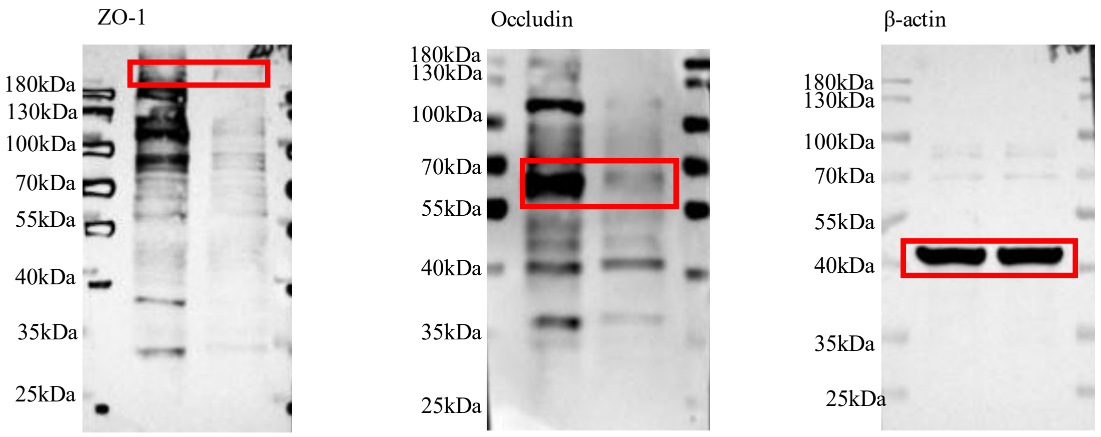

Table S1. Clinical characteristics of NMOSD patients for purified AQP4-IgG

Patient No.	Gender	Serum anti-AQP4 antibody (CBA)
NMOSD-1	Female	1:1000
NMOSD-2	Female	1:320
NMOSD-3	Female	1:320
NMOSD-4	Female	1:3200

CBA, cell-based assay.

Table S2. A list of materials

Category	Source	Identifier
**Antibodies**		
Iba-1	WAKO 1:500 (IF)	Cat#019-19741
Iba-1	Abcam 1:500 (IF)	Cat#ab5076
CD68	Bio-Rad 1:500 (IF)	Cat#1957
AQP4	Proteintech 1:100 (IF)	Cat#16473-1-AP
GFAP	CST 1:400 (IF)	Cat#3670
MAP2	Proteintech 1:200 (IF)	Cat#17490-1-AP
MBP	Millipore 1:200 (IF)	Cat#MAB3869
dMBP	Millipore 1:1000 (IF)	Cat#AB5864
NG2	Millipore 1:200 (IF)	Cat# AB5320
INOS	Abcam 1:100 (IF)	Cat#ab15323
CD16/32	BD biosciences 1:100 (IF)	Cat#553142
CD206	RD system 1:100 (IF)	Cat#AF2535
ZO-1	Thermo Fisher Scientific 1:1000 (WB) 1:100 (IF)	Cat#61-7300
Occludin	Thermo Fisher Scientific 1:1000 (WB)	Cat#71-1500
beta-actin	Proteintech 1:4000 (WB)	Cat#66009-1-Ig
Donkey anti-rabbit Alexa Fluor 488	Jackson Immuno Research	Cat#711-545-152
Cy3-AffiniPure goat Anti-Rabbit	Jackson Immuno Research	Cat#111-165-003
Donkey anti-rat Alexa Fluor 647	Jackson Immuno Research	Cat#712-605-150
Donkey anti-rabbit Alexa Fluor 647	Jackson Immuno Research	Cat#711-605-152
Donkey anti-rat Alexa Fluor 488	Jackson Immuno Research	Cat#712-545-150
Donkey anti-rabbit Alexa Fluor 594	Jackson Immuno Research	Cat#711-585-152
Donkey anti-rat Alexa Fluor 594	Jackson Immuno Research	Cat#712-585-150
HRP Goat Anti-Rabbit IgG (H+L)	Abclonal	Cat#AS014
HRP Goat Anti-Mouse IgG (H+L)	Abclonal	Cat#AS003
**Reagents**		
DMEM/F12	Boster	Cat#PYG0004
DMEM/High Glucose	Boster	Cat#PYG0073
0.25% Tyrisin	Boster	Cat#PYG0015
Fetal Bovine Serum	Boster	Cat#PYG0001

RIPA Lysis buffer	Boster	Cat#AR0102
PMSF	Boster	Cat#AR1178
HEPES powder	Sangon Biotech	Cat#A600264
0.5M EDTA, pH 8.0	Invitrogen	Cat#AM9260G
Cocktail	MCE	Cat#HY-K0010
Protein marker (10-180kDa)	Thermofisher	Cat#26616
5X Loading buffer	Solarbio	Cat#P1040
BCA protein assay kit	Boster	Cat#AR1189
TRIzol	ThermoFisher	Cat#15596026
BODIPY™ FL	ThermoFisher	Cat#D2184
CFSE	Yeasen	Cat#40715ES25
LDL	Guangzhou Yiyuan biotech.Co.Ltd	Cat#YB-001
Dil-LDL	Guangzhou Yiyuan biotech.Co.Ltd	Cat# YB-0011
Evolocumab	Amgen Manufacturing Limited (AML)	/
Cholesterol assay-HDL and LDL/VLDL	Abcam	Cat#ab65390
Neutral red	Sigma	Cat#N4638
LFB staining kit	ServiceBio	Cat#G1030
Oil red O staining	Baso	Cat#BA4081
H-E Stain	Baso	Cat#BA4027
Neutral resins	Baso	Cat#BA7004
PrimeScript™ RT Master Mix	TAKARA	Cat#RR036A
Hieff® qPCR SYBR Green Master Mix	Yeasen	Cat#11201ES03
Pooled Human Complement Serum	Innovative research	Cat#ICSER10ML
R-PLEX Human Neurofilament L Antibody Set	Meso Scale Discovery	Cat#F217X
Isoflurane	RWD Life Science	Cat#R510-22-10
OCT compound	SAKURA	Cat#4583
**Oligonucleotides**		
Primers for qPCR	This paper	Supplementary table 3
**Animals**		
WT C57BL/6 mice	GemPharmatech (Nanjing, China).	Strain NO.N000295

Table S3. A list of primers

Term		Gene	Sequence (5'-3'; Forward - Reverse)
Glycolysis		Pfkl	GAACTACGCACACTTGACCAT-CTCCAAAACAAAGGTCCTCTGG
		Gpi-1	AGGAGTGGTTTCTCGAAGCG-TTTCACTTTGGCCGTGTTCG
		Pkm	CACCCTGGACAACGCTTACA-CCATTCTCCACCTCCGTCAC
		Ogdh	ATCAACCGTGTGACCGACAG-TTGTGTAGGACGGCAAGTCG
OXPHOS		COX6c	ACAGATGCGTGGTCTTCTGG-ACGCCTTCTTTCTTGGCTCAG
		COX7a2	GCTGGCCCTTCGTCAGATT-GGCATCCCATTATCCTCCTGAA
		COX7b	TTGCCCTTAGCCAAAAACGC-TCATGGAAACTAGGTGCCCTC
		NDUFS4	CCGTCTGTAGAGTTCCATCCA-CTGCATGTTATTGCGAGCAGG
Inflammation		Inos	GGAGTGACGGCAAACATGACT-TCGATGCACAACTGGGTGAAC
		Il-6	CTGCAAGAGACTTCCATCCAG-AGTGGTATAGACAGGTCTGTTGG
		IL-1β	TGTCTTGGCCGAGGACTAAGG-TGGGCTGGACTGTTTCTAATGC
		MHC-II	GCGACGTGGGCGAGTACC-CATTCCGGAACCAGCGCA
Cholesterol efflux		APOC1	TGGAGAGCATACCGGATAAACT-AGGAGAACGTGGTCTTCAACT
		NPC2	CCGGTGAAGAATGAATACCC-TTCTTTTTGTCATCTTCAAGTTTCC
		ABCG1	TCTTTGATGAGCCCACCAGT-GGGCCAGTCCTTTCATCA
Remyelination		IGF1	AAATCAGCAGCCTTCCAACTC-GCACTTCCTCTACTTGTGTTCTT
		PDGFα	TGTGCCCATTCGCAGGAAG-GAGGTATCTCGTAAATGACCGTC
Internal reference		β-actin	TGGAATCCTGTGGCATCCATGA-AATGCCTGGGTACATGGTGGTA

RNA data will be made available on request and RNA sequences can be found in the online PrimerBank database: (https://pga.mgh.harvard.edu/primerbank/).
